# A Hybrid MADM Model for Newly Graduated Nurse's Competence Evaluation and Improvement

**DOI:** 10.1155/2021/6658538

**Published:** 2021-03-08

**Authors:** Fengmin Cheng, Yanjun Jin, Ching-Wen Chien, Lei Xiong, Yen-Ching Chuang

**Affiliations:** ^1^Taizhou Hospital of Zhejiang Province Affiliated to Wenzhou Medical University, Taizhou, China; ^2^Institute for Hospital Management, Tsinghua University, Shenzhen Campus, Beijing, China; ^3^School of Architecture and Applied Art, Guangzhou Academy of Fine Arts, Guangzhou, China; ^4^Institute of Public Health & Emergency Management, Taizhou University, Taizhou, China

## Abstract

Nursing departments in hospitals must evaluate the practical competency of newly graduated nurses and assist them to increase their competence. Competency assessments often consider multiple qualitative attributes and use expert knowledge as the basis for decision-making. This study proposes a hybrid multiple attribute decision-making (MADM) model that determines practical competency of the newly graduated nurse as an evaluation framework. A causal influence-network diagram (CIND) and influential weights are obtained from nursing experts' clinical experience using the decision-making trial and evaluation laboratory (DEMATEL)-based analytical network process analysis (DANP). The MOORA-AS method is then used to evaluate the ability expectation ratio-gap for newly graduated nurses in practice. The CIND is used to allow each newly graduated nurse to reduce the performance ratio-gaps between the current level and the aspirational level from a systematic perspective. The empirical data applies to a third-class and a first-class hospital in China. The results show that the proposed hybrid MADM model has reliable results and allows nursing department decision-makers/managers to easily evaluate and systematically improve competencies for newly graduated nurses.

## 1. Introduction

The 2019 Coronavirus (COVID-19) pandemic has had a devastating impact on a global scale on the public health, the economy, the labor market, and other aspects of personal and social life [1]. During global health emergencies, nurses constitute the largest professional group for the provision of first-line healthcare [2, 3]. The clinical nursing and management abilities of nurses are key to identifying, isolating, and managing COVID-19 patients and to providing support for non-COVID-19 patients [3, 4]. 2020 has been named the International Year of the Nurse and Midwife by World Health Organization (WHO) [5].

Resignations among nurses are increasingly common [6]. This fact is worrying because high turnover rates lead to a shortage of nurses and reduce the quality of care, which can lead to adverse patient outcomes [7, 8]. However, more than 20% of all newly graduated nurses leave their jobs within one year, which exacerbates the shortage of nurses [7]. 27.6% of all newly graduated nurses in the United States left their jobs in 2019 [9]. Some studies list the factors that influence the intention of newly graduated nurses to leave their jobs, including working environment, clinical ability and support level, effective leadership, team cohesion, job satisfaction, job stress and coping with self-efficacy, rewards and recognition, and professional development opportunities [7, 10, 11]. Poor ability is one of the main reasons for intended and actual resignations among newly graduated nurses [11, 12]. From the outbreak of Severe Acute Respiratory Syndrome (SARS) in 2003 until the global epidemic involving COVID-19 2019, the increase in the risk of nosocomial infection, the change in nursing requirements, and the increased difficulty of the job increase the willingness of new graduates to leave their profession.

In many cases, newly graduated nurses have insufficient knowledge and experience to supplement their expected job responsibilities, and their knowledge and experience are insufficient to follow practice [13, 14]. This occurs because the profession cannot fully identify and integrate nursing competencies and instill them in the early practice for newly graduated nurses [7]. Practical projects and processes that allow newly graduated nurses to progress have been extensively studied [15–17]. However, the level of competency for newly graduated nurses after graduation that meets the needs in practice is unclear [18], so nursing management faces new challenges.

One study systematically reviewed the assessment of clinical nursing competence and found that the definition of competence is vague and lacks the reliability and validity of systematic measurement tools or strategies [19]. In terms of evaluating competency, studies use multiple attribute decision-making (MADM) methods to evaluate personnel, for selection and for improvement. Chen et al. [20] used the analytical hierarchy process (AHP) to evaluate human resources performance in the logistics market. De Moura and Sobral [21] used the elimination and choice translating reality (ELECTRE) method to classify the competency of employees in a call center. Some MADM methods use interdependence relation perspective, such as the decision-making trial and evaluation laboratory (DEMATEL) [22], the analytical network process analysis (ANP) [23, 24], and the DEMATEL-based analytical network process analysis (DANP) [25]. MADM methods are used for decision-making processes to evaluate personnel, but no studies evaluate the ability of new nurses.

This study develops a hybrid MADM model. The DMATEL method is used to confirm the interaction between attributes and to determine the causal relationship. The results are visualized using a plot, called a causal influence-network diagram (CIND). The influence weights of interdependence between attributes are then derived using the DANP method. The MOORA-AS method is used to evaluate the practical competency of three newly graduated nurses and to determine the attribute of the largest aspirated ratio-gap. The CIND is then used to decrease the maximum aspirated ratio-gap for each newly graduated nurse, and the improvement strategy is based on the system perspective. The hybrid MADM model makes the following contributions to this field of study:

- The CIND visualizes the influential relationship structure within attributes and identifies the key factors among abilities from a systematic perspective in the real world.
- The CIND is used as an improvement tool with a systematic perspective to allow nursing department decision-makers/managers to propose causal improvement suggestions that correspond to each newly graduated nurse's competency. This improvement strategy also avoids the problem of “treating the head when the head aches and treating the foot when the foot hurts” [26].
- The concept of an aspiration level is used to prevent “choosing good apples from rotten apples,” which is a feature of the original MOORA method [27].

The MADM methodology for this study allows nursing researchers to apply various MADM methods to future nursing-related management decision-making problems. The nursing departments of a third-class and first-class hospital in China are used as an empirical case. The results show that the model allows nursing department decision-makers/managers to propose appropriate strategies and improvements to prepare newly graduated nurses for practice.

The remainder of this paper is divided into five sections.  Section 2 details the competency framework for a newly graduated nurse and the use of MADM models to evaluate the competence of personnel and the background of the MADM methods for the proposed model.  Section 3 presents the hybrid MADM model and its corresponding approaches.  Section 4 describes the computational process for the empirical case using the proposed model.  Section 5 discusses the results of Section 4. Finally, the conclusion is presented in Section 6.

## 2. Literature Review

Evaluating the competence of a newly graduated nurse is a personnel assessment problem that involves MADM. It often involves intangible factors and is based on the practical experience of domain-experts. The competency framework for a newly graduated nurse is firstly described. The current use of MADM models for personnel assessment is also discussed. The MADM methods for the proposed hybrid MADM model are then detailed.

### 2.1. The Competency Framework for a Newly Graduated Nurse

Practice readiness refers to the comfort level of new graduates in assuming the professional duties of nurses (professional abilities) [28]. It also refers to thinking like a nurse (professional ability) [29]. The ability index system of a newly graduated nurse is based on the results of the conceptual analysis methods of Mirza et al. [30]. This study uses several databases (CINAHL, PubMed, EBM, and ProQuest) and adopts Rodgers' evolutionary method of conceptual analysis to combine the results of these databases into a conceptual model for the practical preparation of newly graduated nurses. A newly graduated nurse who is preparing to become a qualified nurse must demonstrate cognitive capability (*C*_1_), clinical capability (*C*_2_), and professional capability (*C*_3_) as main competency characteristics. These competencies are the abilities that a newly graduated nurses needs before undertaking professional duties. Nurses must also be able to cope with new and unfamiliar nursing situations so they must evaluate and improve existing abilities. These cognitive, clinical, and professional capabilities contribute to a newly graduated nurse's sense of self-efficacy and a sense of practice readiness [31]. These three main capabilities are described in detail in Table 1.

### 2.2. The MADM Studies Involving Newly Graduated Nurses

In the past few decades, the MADM methods have been widely applied to evaluate and improve personnel competency. Studies that use expert knowledge as a decision-making basis can be roughly divided into three categories: (1) crisp MADM methods, (2) uncertain MADM methods, and (3) other advanced hybrid methods.

Crisp MADM methods. This category of research establishes the function of a decision-making model (i.e., selection or improvement) and uses different decision-making viewpoints, such as preference relationship or influence relationship, so it is a crisp MADM model. For example, Chen et al. [20] used the AHP method to evaluate human resources performance in the logistics market. De Moura and Sobral [21] used the ELECTRE method to classify the competency of employees in a call center. El-Santawy and Ahmed [42] addressed the personnel selection problem using the SDV-MOORA method. The basis of these MADM models is independent relationships, which is inconsistent with the actual situation. Studies regard interdependence as the premise for modeling. Ishizaka and Pereira [23] described a management system that uses MADM analysis and visualization technology, including ANP and PROMETHEE methods. Kucukaltan et al. [24] developed a decision model to identify and prioritize key factors in the logistics industry using a balanced scorecard (BSC) and ANP methods. Aksakal and Dağdeviren [22] developed an integrated approach for personnel selection using DEMATEL and ANP methods. This type of studies establishes the functions of the decision-making model and does not consider the fuzziness of human semantic expression.

Uncertain MADM methods. These consider various ambiguous phenomena and extend the decision-making function using crisp MADM models for the fuzzy expression of human semantics. These are termed fuzzy MADM models. Skrzypek and Dąbrowski [43] used the fuzzy AHP approach to organize an effective team from a group of employees. Karabasevic et al. [44] proposed an uncertain personnel selection framework using SWARA and ARAS methods. Gilan et al. [45] used an interval type-2 fuzzy set for selection of personnel in construction companies using a hierarchy of competency. Wu and Wang [46] combined a cross-entropy and neutrosophic set for the selection of middle-level managers. Canós et al. [47] used fuzzy set methods for personnel selection by evaluating competencies. This type of studies takes into account the ambiguity of human semantic expression, so the decision model uses an independent relationship perspective.

Other advanced hybrid methods. These studies solve complex decision-making problems using a combination of methods, including mathematical methods, soft computing, and machine learning methods. The models are also called advanced hybrid decision-making models. Patel and Jha [48] used an artificial neural network (ANN) to evaluate and predict the work behavior of employees at construction projects. Ahmed et al. [49] used a fuzzy inference system (FIS) to evaluate employee performance. Hanna et al. [50] proposed a general mathematical formula to reliably weigh the capabilities of different project managers in the construction industry. Bohlouli et al. [51] developed a competency evaluation model as a human resource management expert system using mathematical methods. For this type of study, mathematical methods can only process quantitative data. Data mining then requires a large amount of survey data.

### 2.3. DEMATEL-Based ANP Method

The decision-making trial and evaluation laboratory (DEMATEL) is a structural analysis technique that was developed by Battelle Memorial Institute to solve complex structural problems of society in the real world [52, 53]. The results for the DEMATEL method allow decision-makers to understand the contextual and interdependent relationships between elements in the system effectively [54]. Lee et al. [55] extended its application value and further proposed a new weight analysis method, which is based on the concept and calculation of the ANP method [56], namely, the DEMATEL-based ANP or DANP method. The DANP method provides the causal influence-network diagram (CIND) and influence weight from a systematic perspective. Now it is used for various decision-making problems, such as cloud service applications [57], workload stress problems [58], and design of public open space [59].

### 2.4. MOORA-AS Method

MOORA began as an evaluation method that is stable. It was developed by Brauers and Zavadskas [60]. This method combines two different analytical viewpoints: the ratio system and the reference point. The former represents the overall performance of an evaluation system, which allows decision-makers understand the performance of all alternatives. The latter represents the worst performance for attributes in the system. The system is stable in terms of performance ranking, so it is also used for supplier selection [61], failure prioritization [62], and financial services [63]. Liou et al. [64] further integrated the concept of an aspiration level to solve the defect of the reference point in the original MOORA method (i.e., picking a good apple from a group of rotten apples), which is the MOORA-AS method. The MOORA-AS method gives robust results in terms of solving the ranking and selecting alternatives but also reduces the performance ratio-gap for each attribute, so it is close to zero and reaches the aspiration level.

## 3. The Hybrid MADM Model

**Figure 1:**
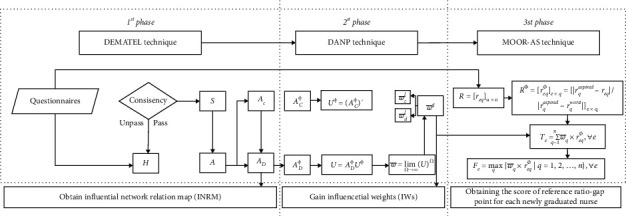
The three main stages of the hybrid MADM model.

The proposed newly graduated nurse's competence framework (as shown in Table 1) is used to develop a hybrid MADM evaluation model, which uses the clinical experience of nursing experts (i.e., administrators, head nurses, and instructors of nurses), the interdependent relationship structure, and the weight between attributes in the real world. The modeling process for the proposed hybrid MADM model uses the DEMATEL, DANP, and MOORA-AS methods. There are three main modeling stages, as shown in Figure 1.

The first stage. The DEMATEL method is used to construct the interdependent relationship structure between attributes.

This stage derives the total influence matrix and the causal influence-network diagram (CIND) between attributes using the clinical experience of an expert group. The computing process for this method follows the following steps:

Step 1: constructing an initial influence relation matrix **H** using clinical nursing experience.
Using the competency framework with *n* attributes, clinical nursing experts use the five-point response scale with language variables (as shown in Table 2) to respond in pairs to the initial influence relation degree between attributes to obtain a nonnegative initial influence relation matrix **H**=[*h*_*ij*_]_*n*×*n*_, as shown in equation (2).
  (1) $$\begin{array}{c} H={\left[h_{ij}\right]}_{n\times n}={\left[\frac{1}{K}\sum_{k=1}^{K}o_{ij}^{k}\right]}_{n\times n},\;i,j\in \left\{1,2,\ldots ,n\right\}, \end{array}$$
where *k* represents the number of clinical nursing experts and *o*_*ij*_^*k*^ represents the initial influence relation matrix for the *k*-th expert.
Step 2: calculating the normalized influence relation matrix **S**.
Before calculating each subsequent degree of influence for matrix **H**, the influence boundary/limited scope is defined and converted into the matrix **S**, as shown in equations (2) and (3).
  (2) $$\begin{array}{c} S={\left[s_{ij}\right]}_{n\times n}={\left[\frac{h_{ij}}{\varepsilon }\right]}_{n\times n}, \end{array}$$
  (3) $$\begin{array}{c} \varepsilon =\max{\left[\left(\underset{1\leq j\leq n}{\max}\sum_{j=1}^{n}h_{ij},\underset{1\leq i\leq n}{\max}\sum_{i=1}^{n}h_{ij}\right)\right]}_{n\times n}, \end{array}$$
where the values in matrix **S** are between 0 and 1 and the maximum sum of a row or a column is always equal to 1.
Step 3: exporting the total influence relation matrix **A**.
Under the condition of matrix **S**, the influence relation degree (direct and indirect) between attributes is continuously calculated using the Markov chain process. If lim_Ω⟶*∞*_ **S** ^Ω^=[0]_*n*×*n*_, the total influence relation matrix is obtained, as shown in equation (4).
  (4) $$\begin{array}{c} A=S^{1}+S^{2}+\cdots +S^{\Omega },\;\text{when }\underset{\Omega ⟶\infty }{\lim}S^{\Omega }={\left[0\right]}_{n\times n}, \end{array}$$
where **I** is an *n* × *n* identity matrix.
Step 4: creating the causal influence-network diagram (CIND).

Using matrix **A**, four different influence indices are created for each attribute. The equations are shown as (5)–(8), and their corresponding explanations are as follows:

Given influence:

(5) $$\begin{array}{c} p_{i}={\left(p_{i}\right)}_{n\times 1}={\left[\sum_{j=1}^{n}a_{ij}\right]}_{n\times 1}. \end{array}$$

Received influence:

(6) $$\begin{array}{c} y_{i}={\left(y_{i}\right)}_{n\times 1}={\left(y_{j}\right)}_{1\times n}^{\Gamma }={\left[\sum_{i=1}^{n}a_{ij}\right]}_{1\times n}^{\Gamma }. \end{array}$$

Prominence:

(7) $$\begin{array}{c} p_{i}+y_{i}. \end{array}$$

Relation:

(8) $$\begin{array}{c} p_{i}-y_{i}, \end{array}$$

where *i*, *j* ∈ {1,2,…, *n*}; let *i*=*j*; superscript Γ is denoted transpose.

The “given influence (*p*_*i*_)” is the total influence degree of the *i*-th attribute on other attributes; otherwise, the “received influence (*y*_*i*_)” is the total influence degree of the *i*-th attribute due to other attributes. “Prominence (*p*_*i*_+*y*_*i*_)” is the total strength of the given and received influence for the *i*-th attribute in a clinical nursing environment. “Relation (*p*_*i*_ − *y*_*i*_)” describes the properties of the main influence of the *i*-th attribute in the clinical nursing environment. If the value of*p*_*i*_ − *y*_*i*_ is positive, then the *i*-th attribute affects other attributes. If the value is negative, the *i*-th attribute is influenced by other attributes. A causal influence-network diagram (CIND) is created using “prominence (*p*_*i*_+*y*_*i*_)” and “relation (*p*_*i*_ − *y*_*i*_).”

The second stage: the DANP method is used to derive the influential weight using the interdependent relationship structure between attributes.

The DANP method is used to derive the influential weights from the results for the total influence relation matrix **A** using the DEMATEL method. If the capability framework is hierarchical, matrix **A** has two levels: the matrix with attribute level **A**_*C*_ and aspect level **A**_*D*_. The computing process for this method follows the following steps:

Step 1: transferring the unweighted supermatrix **A**_*C*_^Φ*∗*^ from the total influence relation matrix **A**_*C*_.
The proportion and transposition of influence relations at different aspect levels mean that this conforms to the ANP method calculation process. The normalized total influence relation matrix **A**_*C*_^Φ^, which is also known as unweighted supermatrix **A**_*C*_^Φ*∗*^, is shown in
(9)
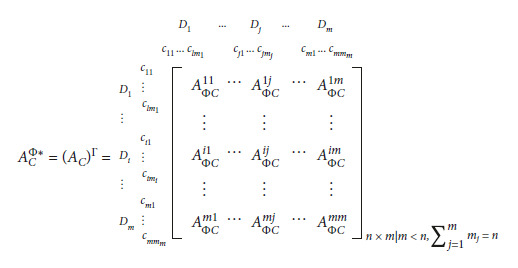

where **A**_*C*_^Φ^ represents the normalized influence relation ratio for matrix **A**_*C*_ within the aspect **A**_*D*_, superscript Γ is the transpose, *n* represents the number of attributes, and *m* represents the number of aspects.
For this step, the normalization action is explained using the submatrix **A**_Φ*C*_^11^ in the matrix **A**_*C*_^Φ^ as the sample, as shown in equation (10). Other submatrices **A**_Φ*C*_^*mm*^ are then used to establish a complete unweighted supermatrix by the same actions.
(10)
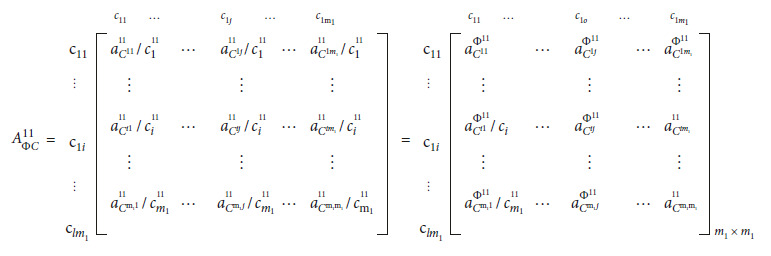

where *c*_*i*_^11^=∑_*j*=1_^*m*_1_^ *a*_*C*^*ij*^_^11^, *i*=1,2,…, *m*_1_.
Step 2: constructing a weighted supermatrix **U** using different influence ratio perspectives.
The unweighted supermatrix **A**_*C*_^Φ*∗*^ only converts numerical values into the proportional form and does not consider the difference in influence proportion between aspects (i.e., the proportion of each aspect is equal). In this step, the total influence relation matrix **A**_*D*_ is the coefficient (i.e., transposed and normalized matrix **A**_*D*_^Φ*∗*^) to adjust the matrix **A**_*C*_^Φ*∗*^, so the final influence weight is closer to reality. In terms of ideas and methods. It also addresses the defects of the weighted super-matrix for the original ANP method. The equations are
  (11) $$\begin{array}{c} A_{D}^{\Phi *}={\left(A_{D}^{\Phi }\right)}^{\Gamma }=\left[\begin{array}{ccccc} \frac{a_{11}^{D_{11}}}{d_{1}} & \cdots & \frac{a_{1o}^{D_{1j}}}{d_{1}} & \cdots & \frac{a_{1m}^{D_{1m}}}{d_{1}}\\ \vdots & & \vdots & & \vdots \\ \frac{a_{i1}^{D_{i1}}}{d_{i}} & \cdots & \frac{a_{ij}^{D_{ij}}}{d_{i}} & \cdots & \frac{a_{im}^{D_{im}}}{d_{i}}\\ \vdots & & \vdots & & \vdots \\ \frac{a_{m1}^{D_{m1}}}{d_{m}} & \cdots & \frac{a_{mj}^{D_{mj}}}{d_{m}} & \cdots & \frac{a_{mm}^{D_{mm}}}{d_{m}} \end{array}\right]={\left[\begin{array}{ccccc} a_{11}^{\Phi_{11}} & \cdots & a_{1j}^{\Phi_{1j}} & \cdots & a_{1m}^{\Phi_{1m}}\\ \vdots & & \vdots & & \vdots \\ a_{i1}^{\Phi_{i1}} & \cdots & a_{ij}^{\Phi_{ij}} & \cdots & a_{im}^{\Phi_{im}}\\ \vdots & & \vdots & & \vdots \\ a_{m1}^{\Phi_{m1}} & \cdots & a_{mj}^{\Phi_{mj}} & \cdots & a_{mm}^{\Phi_{mm}} \end{array}\right]}_{m\times m}, \end{array}$$
where *d*_*i*_=∑_*j*=1_^*m*^ *a*_*ij*_^*Di*  *j*^, *i*=1,2,…, *m*.
  (12) $$\begin{array}{c} U=A_{D}^{\Phi *}\times A_{C}^{\Phi *}={\left[\begin{array}{ccccc} a_{11}^{\Phi_{11}}\times A_{\Phi C}^{11} & \cdots & a_{i1}^{\Phi_{i1}}\times A_{\Phi C}^{i1} & \cdots & a_{m1}^{\Phi_{m1}}\times A_{\Phi C}^{m1}\\ \vdots & & \vdots & & \vdots \\ a_{1j}^{\Phi_{1j}}\times A_{\Phi C}^{1j} & \cdots & a_{ij}^{\Phi_{ij}}\times A_{\Phi C}^{ij} & \cdots & a_{mj}^{\Phi_{mj}}\times A_{\Phi C}^{mj}\\ \vdots & & \vdots & & \vdots \\ a_{1m}^{\Phi_{1m}}\times A_{\Phi C}^{1m} & \cdots & a_{im}^{\Phi_{im}}\times A_{\Phi C}^{im} & \cdots & a_{mm}^{\Phi_{mm}}\times A_{\Phi C}^{mm} \end{array}\right]}_{n\times n|m<n,\sum_{j=1}^{m}m_{j}=n}. \end{array}$$
Step 3: calculating the influential weights.

The weighted supermatrix **U** is multiplied by itself several times to become a steady-state supermatrix using the Markov chain concept and calculation. The limited supermatrix *ϖ* is then obtained, as shown in

(13) $$\begin{array}{c} \varpi =\underset{\Omega ⟶\infty }{\lim}{\left(U\right)}^{\Omega }. \end{array}$$

The third stage: the MOORA-AS method is used to calculate the performance ratio-gaps on competencies for newly graduated nurses.

The MOORA-AS method is used to calculate the total competency performance ratio-gap and the ratio-gap for each attribute for newly graduated nurses. The steps for this method are as follows:

Step 1: Establishing a performance matrix for newly graduated nurses.
The performance matrix **R** uses the evaluation framework (as shown in Table 1), whereby clinical nursing supervisors or managers use an 11-point scale (0 is the worst; 10 is the best) to evaluate the ability of newly graduated nurses, and the ability of each new nurse is calculated using the average method. The performance matrix **R** is defined in
  (14) $$\begin{array}{c} R={\left[r_{\text{eq}}\right]}_{u\times n},\;e\in \left\{1,2,\ldots ,u\right\},j\in \left\{1,2,\ldots ,n\right\}, \end{array}$$
where *u* represents the number of newly graduated nurses and *n* represents the number of attributes.
Step 2: normalizing the performance matrix using the aspiration level.
In MADM decision-making problems, the scale for each attribute often lies within a known range, from the maximum value to the minimum value of the scale. When an aspiration level is used, the maximum value for the scale becomes the aspirated solution (i.e., *r*_*q*_^aspired^). However, the minimum value is the worst solution (i.e., *r*_*q*_^worst^). The normalized matrix is shown in
  (15) $$\begin{array}{c} R^{\Phi }={\left[r_{\text{eq}}^{\Phi }\right]}_{e\times q}={\left[\frac{\left|r_{q}^{\text{aspired}}-r_{\text{eq}}\right|}{\left|r_{q}^{\text{aspired}}-r_{q}^{\text{worst}}\right|}\right]}_{e\times q}, \end{array}$$
where *r*_eq_^Φ^ represents the performance gap ratio between 0 and 1.
Step 3: building the score for the ratio-gap system for each newly graduated nurse.
The ratio-gap system for newly graduated nurses reflects the overall gap between current performance and aspirational level. The lower the value, the higher the ranking for the new nurse in terms of all new nurse candidates. The calculation is shown as
  (16) $$\begin{array}{c} T_{e}=\sum_{q=1}^{n}\varpi_{q}\times r_{\text{eq}}^{\Phi },\;\forall e, \end{array}$$
where *ϖ*_*q*_ represents the influential weights, as calculated using the DANP method.
  Step 4 : Calculating the score for the reference ratio-gap point for each newly graduated nurse.

The reference point for newly graduated nurses is the worst attribute (the maximum ratio-gap), so this is the maximum ratio-gap for ability, as shown in

(17) $$\begin{array}{c} F_{e}=\underset{q}{\max}\left\{\varpi_{q}\times r_{eq}^{\Phi }|q=1,2,\ldots ,n\right\},\;\forall e. \end{array}$$

## 4. Empirical Case Study

A third-class and first-class official hospital in China are used to illustrate the use of the hybrid proposed MADM model to evaluate the ability and determine the improvements in newly graduated nurses.

### 4.1. The Background Description and Data Collection

The case involves a third-class and a first-class hospital that offer medical treatment, scientific research, teaching, and prevention. In terms of medical disciplines, the case hospital has 14 provincial specialized disease centers in the region (there are 20 centers in the region) and 23 key medical disciplines (orthopedics, digestive medicine, and cardiovascular medicine). There are 1,924 beds in the case hospital and an average of 110,000 hospitalizations each year. In the 2019 self-assessed statistical report, the number of outpatients was over 2.7 million, and the number of discharged patients was about 108,000. The number of medical disciplines and the population in the region mean that the case hospital is one of the major hospitals in the region.

Every year, the hospital employs approximately 150 new nurses. Every month, the hospital gives a series of standardized training courses to track the training and assessment results for new nurses. There is also a series of assessment standards for clinical practice. These measures allow new nurses to integrate into clinical nursing work as soon as possible and provide better nursing services for hospitalized patients. The evaluation of competency includes quantitative indicators and nonquantitative indicators; therefore, in addition to the existing clinical quantitative index evaluation system, the decision-making model for competency allows nursing departments to systematically evaluate the ability of new nurses and to propose improvement strategies, which is the most important management problem in hospitals at present.

This study proposes a hybrid MADM model that uses the newly graduated nurse competency framework (as shown in Table 1) from a past study and MADM methods. For this study, data was collected from 15 clinical nursing experts (2 nursing administrators, 6 head nurses, and 7 nursing instructors). In terms of clinical nursing experience, one expert had more than 2 to 3 years, 1 expert had more than 4 to 5 years, 3 experts had more than 5 to 10 years, and 10 experts had more than 10 years. These head nurses or nurse instructors are nurses with much professional and clinical experience in nursing. They are also aware of the relationship between the abilities that newly graduated nurses should possess and these abilities. Three newly graduated nurses with less than 3 years of employment are the evaluation objects.

### 4.2. Calculating the Interdependence Relationship between Attributes Using the DEMATEL Method

Based on the practical experience of 15 clinical nursing experts, an initialization influence relationship matrix was established, as shown in Table 3. The quality of this matrix is very important because it affects the results for the structure of a causal relationship and the weight, as well as subsequent evaluation and improvement strategies that are proposed as a consequence. Using the concept of a confidence level and a 15-fold cross-validation process, the average variance consistency for the initialization matrix is between 0.043 (i.e., 4.3%) and 0.011 (i.e., 1.1%), with an average of 0.019 (i.e., 1.9%, less than 5%). The significant confidence level is 98.1% (i.e., greater than 95%), so the experience of this group of clinical nursing experts is consistent, and the subsequent results are very stable, as shown in Table 4.

The initialization matrix **H** describes the total influence relationship degree between attributes using equations (2) to (4), which is the total influence relationship matrix **A**, as shown in Table 5.

Finally, the total influence relationship matrix **A** uses (5) to (8) to calculate the total “given influence (*p*_*i*_)” and “received influence (*y*_*i*_)” for each attribute and the “prominence (*p*_*i*_+*y*_*i*_)” and “relation (*p*_*i*_ − *y*_*i*_),”as shown in Table 6.

**Figure 2:**
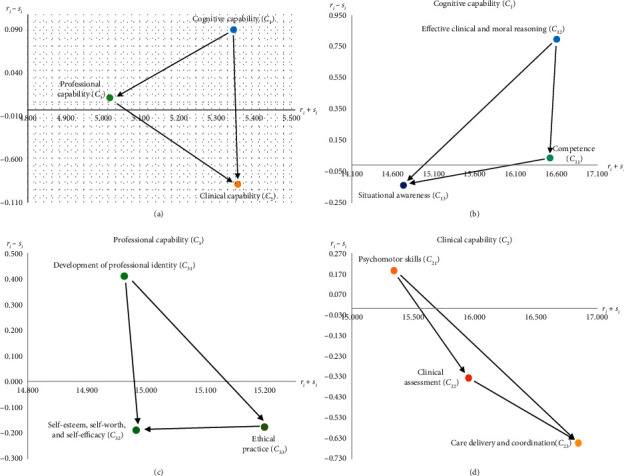
(a) Newly graduated nurse competency framework. (b) Cognitive capability (*C*1). (c) Professional capability (*C*3). (d) Clinical capability (*C*2).

A causal influence-network diagram (CIND) allows nursing department decision-makers/managers to understand the interaction between all aspects and attributes for newly graduated nurses by identifying the influence structure between “prominence (*p*_*i*_+*y*_*i*_)” and “relation (*p*_*i*_ − *y*_*i*_),”as shown in Figure 2.

In Figure 2, in terms of aspect level, “cognitive capability (*C*_1_)” is the basis for the other two abilities (“professional capability (*C*_3_)” and “clinical capability (*C*_2_)”). Only when a newly graduated nurse has a clear understanding and judgment of situations and surrounding things in the clinical environment, are the advantages, disadvantages, and limitations obvious, and only then can professional values, beliefs, and goals be defined. This develops a strong internal driving force to continuously work to achieve goals. Under the influence of this internal drive, nurses study hard and improve their self-competence, professional ability, and clinical ability. “Effective clinical and moral reasoning (*C*_12_),” “psychomotor skills (*C*_21_),” and “development of professional identity (*C*_31_)” are the most critical attributes at the corresponding levels.

### 4.3. Calculating the Weights of Influence Relationship for Attributes Using DANP

The total influence relation matrix**A**describes the influence weight for each attribute using (9) to (13), which also shows the importance of each attribute to the overall ability of newly graduated nurses, as shown in Table 7. In terms of weight, an improvement in “clinical capability (*C*_2_)” is the premise for newly graduated nurses to be better qualified for clinical nursing work. If there is a rapid improvement in clinical ability, newly graduated nurses can continuously improve their ability to observe and discover problems and their prospective analysis ability, so newly graduated nurses can learn to make correct clinical judgments and decisions and address existing or potential clinical problems. In the process of clinical nursing practice, clinical ability is the most important of these three competencies at aspect level.

### 4.4. Evaluating and Ranking Newly Graduated Nurses Using the MOORA-AS Method

Three newly graduated nurses were assessed by clinical nursing experts using the newly graduated nurse's ability assessment framework (as shown in Table 1) and an 11-point scale (the worst is 0 points; the best is 10 points) to derive an original performance score using the average method. The original performance score uses equations (14) to (17) for the MOORA-AS method to calculate the performance ratio-gaps for the overall ability of the three newly graduated nurses. The results are shown in Table 8. These results show that “newly graduated nurse *B*” has the smallest ratio-gap with the aspirational level, so this is the best of the three newly graduated nurses.

## 5. Discussion

Based on these results, the improvement strategies and the causal relationship are further explained and the original MOORA and MOORA-AS methods are compared.

### 5.1. Systemic Improvement Strategy Using the CIND

**Figure 3:**
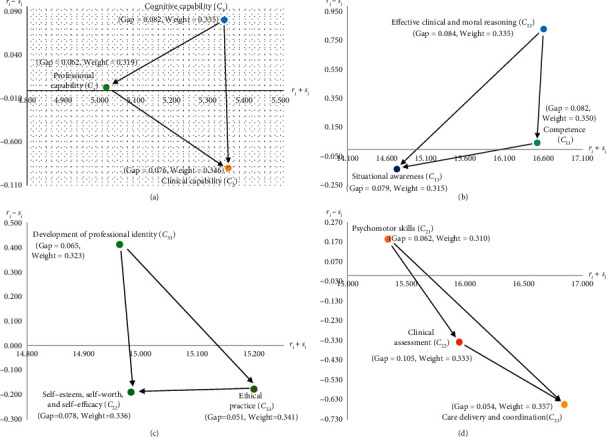
(a) Newly graduated nurse competency framework. (b) Cognitive capability (*C*1). (c) Professional capability (*C*3). (d) Clinical capability (*C*2).

Improvement strategies for newly graduated nurses are formulated using the results for the reference ratio-gap ratio (Table 8), the CIND (Figure 2), and the weight (Table 7). The results for newly graduated nurse *A* are shown in Figure 3. “Cognitive capability (*C*_1_)” is the worst attribute (i.e., ratio-gap = 0. 082) at the aspect level, and it is also the most main influence source for the entire capability framework (relation is ranked first). Further analysis at the attribute level shows that “effective clinical and moral reasoning (*C*_12_)” has the maximum ratio-gap (i.e., 0.084). It is also the main influence source.

Using these results, the nursing department decision-makers/managers might propose appropriate training courses or alternatives to improve “effective clinical and moral reasoning (*C*_12_).” When “effective clinical and moral reasoning (*C*_12_)” is improved, the overall ability of newly graduated nurse *A* will improve. This study shows that the quality of standardized training for nurses after graduation is important. Standardized training for nurses uses the basic stages of the growth for new graduated nurses and remains crucial for a nurse's professional development. It is a process that promotes the formation of professional ethics, cultivation of clinical thinking mode, and standardization and improvement of nursing practice for new nurses.

To improve the performance of newly graduated nurse *A* in terms of the *C*_12_ criteria, a systematic symptom care course for each symptom is necessary: overview; nursing evaluation; nursing measures and case analysis. Newly graduated nurse *A* must attain basic theoretical knowledge and skills for psychological care, health education, and other humanistic care knowledge. Newly graduated nurse *A* must understand the internal relationship between symptoms and disease for symptom analysis, make correct clinical judgments, and better guide clinical practice.

### 5.2. Difference between Original MOORA and MOORA-AS Methods

Table 9 shows the difference between different methods for the same performance data. The ranking for the reference ratio-gap point is different. The ranking for the original MOORA method is newly graduated nurse *A*≻ newly graduated nurse *B*≻ newly graduated nurse *C*, but for the MOORA-AS method, the ranking is newly graduated nurse *B*≻ newly graduated nurse *A*≻ newly graduated nurse *C*. Using the original MOORA method, the attribute ratio-gap has a large number of zero scores or perfect scores for newly graduated nurse *B* and newly graduated nurse *C*, who are the best and worst cases, respectively. Many ratio-gaps = 0 points (i.e., *C*_1_, *C*_11_, *C*_12_, *C*_13_, *C*_21_, *C*_22_, *C*_32_, *C*_33_) for newly graduated nurse *B*, and many proportion-gaps = 1 point (i.e., *C*_1_, *C*_11_, *C*_12_, *C*_13_, *C*_2_, *C*_21_, *C*_22_, *C*_23_, *C*_32_, *C*_33_) for newly graduated nurse *C*. This is because the original MOORA method uses relative concepts for ranking, but MOORA-AS uses absolute concepts (i.e., aspiration level) for ranking. The MOORA-AS method gives a more robust ranking for nursing department decision-makers/managers, especially if the measurement scale is known.

## 6. Conclusions

Professional nurses play a key role in the fight against COVID-19 infection. The shortage of professional nurses has been a problem for the medical system and hospitals because of the high turnover rate for newly graduated nurses. This study proposes a decision-making model to allow nursing department decision-makers/managers to improve the competence of newly graduated nurses and to decrease the ability gaps between newly qualified and professional nurses. A hybrid MADM model is proposed, which uses an evaluation framework that is based on findings for the competency characteristics of new graduate nurses from previous studies, combined with the MADM method, which is based on the system perspective. An empirical study of the hybrid MADM model uses data from a third-class and a first-class hospital in China, including the modeling process, result analysis, and improvement strategy. The highlights of each stage are as follows:

1. The competency framework for this study describes the competency that is required for a newly graduated nurse before entering a new nursing position and the ability to cope with new and unfamiliar nursing situations by evaluating and improving existing competencies. This competency framework is based on the results of previous studies of competency problems involving recent graduates. The problem is a MADM problem and is the subject of few studies.
2. The results for the CIND show that cognitive capability (*C*_1_) is a critical factor for the other two abilities: professional capability (*C*_3_) and clinical capability (*C*_2_). This is because the cognitive capability is improved. The professional capability and clinical capability are also improved to a certain extent. Effective clinical and moral reasoning (*C*_12_), psychomotor skills (*C*_21_), and development of professional identity (*C*_31_) are the most critical attributes at the corresponding levels.
3. The results for influential weight show that an improvement in clinical capability (*C*_2_) is related to newly graduated nurses being better qualified for clinical nursing work, so newly graduated nurses make correct clinical judgments and decisions and finally address the current and potential clinical problems of patients correctly. For the process of clinical nursing practice, the weight for clinical ability is the most important of these three competencies in terms of aspect level.
4. In terms of a systematic improvement strategy for newly graduated nurse *A*, nursing department decision-makers/managers could propose appropriate training courses or programs to improve effective clinical and moral reasoning (*C*_12_) because this attribute has the maximum ratio-gap and is also a critical factor. If an improvement is achieved, other abilities will also be improved.
5. The results for the original MOORA and MOORA-AS methods show that the MOORA-AS method addresses the problem of ranking and is better than the original MOORA method for MADM problems because an absolute concept (i.e., aspiration level) gives more stable results than a relative concept.

This research model allows hospitals to evaluate the ability of newly graduated nurses and to propose improvement strategies from a systematic viewpoint. The nursing department can combine practical training courses to create bespoke training courses for each newly graduated nurse. The results of this study are limited to the experience for a single hospital because different hospitals may have different weights and performance scores for newly graduated nurses. This study does not consider the uncertainty (i.e., fuzzy phenomenon) between competencies in the clinical nursing environment. Future research models might use various other theories and methods (such as intuitionistic fuzzy theory [65], 2-tuple linguistic model [66], and a gray system [67]) to establish decision-making models that have more technical and practical value for newly graduated nurses.

## Figures and Tables

**Table 1:** The competency framework for newly graduated nurses to become practice-ready.

Aspect	Attribute	Description	Ref.
Cognitive capability (*C*_1_)	Competence (*C*_11_)	Newly graduated nurses know how to use nursing knowledge in particular contexts or situations	[32, 33]
	Effective clinical and moral reasoning (*C*_12_)	Newly graduated nurses should exhibit critical thinking and problem-solving skills to make effective clinical judgments in different clinical situations, and they should also be expected to use nursing procedures to plan and evaluate effective nursing measures for patients. Moral reasoning plays a role in the formulation of professional practice.	[28, 29, 34–36]
	Situational awareness (*C*_13_)	Newly graduated nurses should be able to determine their limitations in different clinical situations and seek help when needed. This cognitive ability of situational awareness is crucial to providing safe nursing.	[34, 37]

Clinical capability (*C*_2_)	Psychomotor skills (*C*_21_)	Newly graduated nurses must have the necessary mental skills to be psychologically prepared for practical nursing work	[28, 29, 35, 38]
	Clinical assessment (*C*_22_)	Clinical evaluation is an important skill that reflects clinical ability, including analyzing a patient's condition, detecting changes in a patient's condition, predicting problems, and identifying a patient's needs	[29, 36, 39]
	Care delivery and coordination (*C*_23_)	Soft skills to provide patient care and coordinate and cooperate with colleagues in the workplace, such as effective interpersonal communication, teamwork, and time management, are also basic requirements for providing effective patient care	[28, 36, 38, 40]

Professional capability (*C*_3_)	Development of professional identity (*C*_31_)	The development of the professional identity of newly graduated nurses allows them to fulfil the multiple roles of professional nurses, such as administration, clinical and nursing research	[28, 37]
	Self-esteem, self-worth, and self-efficacy (*C*_32_)	Newly graduated nurses should have self-esteem, self-worth, and the ability to operate independently and be responsible for their nursing practices. The individual's belief in their readiness for practice is also an important element of the various capabilities.	[35–37, 41]
	Ethical practice (*C*_33_)	Newly graduated nurses must also have a moral and ethical code that is based on moral reasoning components (cognitive abilities).	[29]

**Table 2:** Five-point response scale using linguistic variables.

Linguistic variables	Crips number
No influence (NI)	0
Weak influence (WI)	1
Medium influence (MI)	2
Strong influence (SI)	3
Very strong influence (VSI)	4

**Table 3:** Initial influence relation matrix **H**.

Attribute	*C*_11_	*C*_12_	*C*_13_	*C*_21_	*C*_22_	*C*_23_	*C*_31_	*C*_32_	*C*_33_
*C*_11_	0.000	3.000	2.400	2.667	3.067	2.867	2.400	2.267	2.133
*C*_12_	3.133	0.000	2.533	2.067	3.467	3.067	2.733	2.400	2.667
*C*_13_	2.133	2.200	0.000	2.600	2.267	2.733	1.867	2.133	2.133
*C*_21_	2.533	2.200	2.333	0.000	2.000	3.000	2.200	2.600	2.467
*C*_22_	3.067	2.667	1.800	2.000	0.000	2.800	2.200	2.400	2.400
*C*_23_	2.800	2.733	2.733	2.600	2.800	0.000	1.933	2.200	2.400
*C*_31_	2.400	2.200	2.133	2.267	2.333	2.600	0.000	2.533	2.667
*C*_32_	2.133	2.200	2.200	2.533	2.133	2.467	2.400	0.000	2.267
*C*_33_	2.467	2.467	2.267	2.067	2.200	2.533	2.267	2.333	0.000

*Note.* Values are calculated from equation (1).

**Table 4:** The variance of the 15-fold cross-validation.

Number of experts	No. 1	No. 2	No. 3	No. 4	No. 5	No. 6	No. 7	No. 8
Gap	0.016	0.011	0.014	0.014	0.019	0.037	0.023	0.018
Gap (%)	1.6%	1.1%	1.4%	1.4%	1.9%	3.7%	2.3%	1.8%
Number of experts	No. 9	No. 10	No. 11	No. 12	No. 13	No. 14	No. 15	Average
Gap	0.043	0.014	0.017	0.021	0.015	0.016	0.013	0.019
Gap (%)	4.3%	1.4%	1.7%	2.1%	1.5	1.6%	1.3%	1.9%

*Note.* The significant confidence equation is (1/9(9 − 1))∑_*i*=1_^15^∑_*j*=1_^15^(|*h*_*ij*_^15^ − *h*_*ij*_^15−1^|/*h*_*ij*_^15^) × 100%=1.9%<5%; i.e., significant confidence is 98.1%.

**Table 5:** Total influence relation matrix **A**.

Attribute	*C*_11_	*C*_12_	*C*_13_	*C*_21_	*C*_22_	*C*_23_	*C*_31_	*C*_32_	*C*_33_
*C*_11_	0.877	0.959	0.886	0.910	0.986	1.042	0.868	0.896	0.902
*C*_12_	1.048	0.884	0.932	0.930	1.045	1.097	0.921	0.943	0.964
*C*_13_	0.858	0.827	0.692	0.810	0.850	0.923	0.754	0.793	0.802
*C*_21_	0.920	0.873	0.831	0.749	0.887	0.983	0.809	0.854	0.859
*C*_22_	0.945	0.895	0.815	0.836	0.810	0.981	0.813	0.851	0.861
*C*_23_	0.965	0.926	0.876	0.885	0.952	0.901	0.830	0.871	0.889
*C*_31_	0.907	0.865	0.816	0.834	0.891	0.960	0.711	0.844	0.859
*C*_32_	0.866	0.835	0.791	0.816	0.854	0.923	0.782	0.713	0.816
*C*_33_	0.891	0.857	0.804	0.809	0.869	0.938	0.788	0.820	0.734

*Note.* Values are calculated from equations (1) to (4).

**Table 6:** The influence indicators for aspects and attributes.

Aspect	*p*_*i*_	*y*_*i*_	*p*_*i*_+*y*_*i*_	*p*_*i*_ − *y*_*i*_	Attribute	*p*_*i*_	*y*_*i*_	*p*_*i*_+*y*_*i*_	*p*_*i*_ − *y*_*i*_
*C*_1_	2.711	2.627	5.338	0.084	*C*_11_	8.326	8.277	16.603	0.049
					*C*_12_	8.764	7.923	16.687	0.842
					*C*_13_	7.309	7.442	14.751	−0.134

*C*_2_	2.630	2.719	5.349	−0.089	*C*_21_	7.764	7.578	15.342	0.186
					*C*_22_	7.807	8.144	15.951	−0.337
					*C*_23_	8.096	8.749	16.845	−0.653

*C*_3_	2.510	2.505	5.016	0.005	*C*_31_	7.687	7.275	14.962	0.411
					*C*_32_	7.397	7.585	14.982	−0.188
					*C*_33_	7.511	7.687	15.198	−0.176

*Note.* Values are calculated from equations (5) to (8).

**Table 7:** Influential weights of attributes.

Aspect	Local weight	Ranking	Attribute	Local weight	Ranking	Global weight	Ranking
*C*_1_	0.335	2	*C*_11_	0.350	1	0.117	2
			*C*_12_	0.335	2	0.112	4
			*C*_13_	0.315	3	0.105	8

*C*_2_	0.346	1	*C*_21_	0.310	3	0.107	7
			*C*_22_	0.333	2	0.115	3
			*C*_23_	0.357	1	0.124	1

*C*_3_	0.319	3	*C*_31_	0.323	3	0.103	9
			*C*_32_	0.336	2	0.107	6
			*C*_33_	0.341	1	0.109	5

*Note.* Values are calculated using equations (9) to (13).

**Table 8:** Results for the evaluation of newly graduated nurses using the MOORA-AS method.

	Original performance			Local weight	MOORA-AS		
	Newly graduated nurse A	Newly graduated nurse B	Newly graduated nurse C		Newly graduated nurse A	Newly graduated nurse B	Newly graduated nurse C
*C*_1_				0.335	0.082	0.074	0.164
*C*_11_	7.667	8.000	5.500	0.350	0.082	0.070	0.157
*C*_12_	7.500	7.600	5.250	0.335	0.084	0.081	0.159
*C*_13_	7.500	7.800	4.500	0.315	0.079	0.069	0.173
*C*_2_				0.346	0.076	0.067	0.139
*C*_21_	8.000	8.600	7.250	0.310	0.062	0.043	0.085
*C*_22_	6.833	7.400	4.000	0.333	0.105	0.086	0.200
*C*_23_	8.500	8.200	6.750	0.357	0.054	0.064	0.116
*C*_3_				0.319	0.062	0.072	0.104
*C*_31_	8.000	6.400	6.500	0.323	0.065	0.116	0.113
*C*_32_	7.667	7.800	6.500	0.336	0.078	0.074	0.118
*C*_33_	8.500	9.000	7.250	0.341	0.051	0.034	0.094
The ratio-gap system					0.220 (2)	0.212 (1)	0.406 (3)
The reference ratio-gap point					0.082 (2)	0.074 (1)	0.164 (3)
Final rank					2	1	3

*Note.* The values of the MOORA-AS method are weighted and are calculated using equations (14) to (17).

**Table 9:** Results for the original MOORA and the MOORA-AS methods.

Aspect/attribute	Local weight	Original MOORA			MOORA-AS		
		Newly graduated nurse A	Newly graduated nurse B	Newly graduated nurse C	Newly graduated nurse A	Newly graduated nurse B	Newly graduated nurse C
*C*_1_	0.335	0.030	0.000	0.335	0.082	0.074	0.164
*C*_11_	0.350	0.047	0.000	0.350	0.082	0.070	0.157
*C*_12_	0.335	0.014	0.000	0.335	0.084	0.081	0.159
*C*_13_	0.315	0.029	0.000	0.315	0.079	0.069	0.173
*C*_2_	0.346	0.067	0.021	0.346	0.076	0.067	0.139
*C*_21_	0.310	0.138	0.000	0.310	0.062	0.043	0.085
*C*_22_	0.333	0.055	0.000	0.333	0.105	0.086	0.200
*C*_23_	0.357	0.000	0.061	0.357	0.054	0.064	0.116
*C*_3_	0.319	0.042	0.103	0.313	0.062	0.072	0.104
*C*_31_	0.323	0.000	0.323	0.303	0.065	0.116	0.113
*C*_32_	0.336	0.034	0.000	0.336	0.078	0.074	0.118
*C*_33_	0.341	0.097	0.000	0.341	0.051	0.034	0.094
The ratio-gap system		0.139 (2)	0.124 (1)	0.994 (3)	0.220 (2)	0.212 (1)	0.406 (3)
The reference ratio-gap point		0.06 (1)	0.103 (2)	0.346 (3)	0.082 (2)	0.074 (1)	0.164 (3)

*Note.* The values for the original MOORA and MOORA-AS methods are weighted.

## Data Availability

The data used to support the findings of this study are included in the article.
